# Longitudinal examination of a refined four-factor model of Protective Behavioural Strategies: Psychosocial barriers to their use and protective effects on students’ alcohol consumption

**DOI:** 10.1016/j.abrep.2026.100694

**Published:** 2026-04-03

**Authors:** Maëlle Fleury, Jessica Mange, Maxime Mauduy

**Affiliations:** a Laboratoire de Psychologie de Caen Normandie (LPCN UR 7452), Université de Caen Normandie, Caen, France; b Laboratoire de Psychologie Sociale (LPS, UR 4471), Université Paris Cité, Institut de Psychologie, Boulogne-Billancourt, France

**Keywords:** Protective behavioral strategies, Alcohol, Young adults, Psychosocial determinants, Longitudinal design

## Abstract

**Objective:**

Protective Behavioral Strategies (PBS) are cognitive-behavioural strategies that are promising to reduce risky alcohol consumption among students. This research investigates 1) the role of psychosocial determinants of drinking (motives and perceived social norms) as potential barriers to their use, and 2) the protective value of the four‑PBS against risky consumption.

**Methods:**

Two longitudinal studies (two-waves, one-year interval) in French university students were conducted. Latent Change Score Model (*N* = 164) tested how changes in psychosocial determinants were related to within-person changes in four PBS use. Cross-Lagged Panel Model (*N* = 188) examined reciprocal associations between each PBS and typical drinking quantity.

**Results:**

Firstly, modifying the manner of drinking strategy (MOD; i.e., inhibiting risky drinking) showed the largest within-person change over one year, increasing as students grew older and decreased in social motives. Secondly, higher MOD use was consistently associated with fewer drinks per occasion, whereas other PBS domains showed mainly same-wave links with drinking. Prior drinking also strongly predicted later consumption, indicating that MOD operates as a protective factor beyond the relative stability of students’ drinking patterns.

**Conclusions:**

Findings highlight MOD strategy as a priority target for reducing student alcohol consumption. Strengthening MOD may help reduce typical number of drinks, but interventions must also tackle social drinking motives that discourage its use. Student-focused prevention programmes could therefore prioritise presenting MOD as socially acceptable and compatible with enjoyable nights out, helping students to preserve social connection and pleasure while shifting towards safer, more sustainable patterns of alcohol use.

## Highlights

• Protective Behavioural Strategies help reduce risky alcohol use in students.
• Two-wave LCSM and CLPM examine links between PBS, motives, norms and drinking.
• Higher MOD use predicts fewer drinks per occasion one year later in students.
• Increases in social drinking motives predict decreases in MOD use over time.
• Prior drinking remains the strongest predictor of later alcohol consumption.

## 1 Introduction

For years, alcohol has remained the most widely consumed psychoactive substance in France (Spilka et al., 2024) and elsewhere (SAMHSA, S. A. and M. H. S. A. , 2025, Sudhinaraset et al., 2016). Despite various prevention efforts, episodic heavy drinking patterns such as binge drinking have become a specific focus in recent decades, particularly among young adults who seek rapid and heavy intoxication. Because of the various issues of these new practices on cognitive functions and social and academical spheres (Bonnaire et al., 2022, Gierski et al., 2018, Townshend et al., 2014), research has focused on prevention, especially targeting risky drinking patterns and their consequences. Within this perspective, alcohol-related Protective Behavioural Strategies (PBS), have emerged as promising tools (Grazioli et al., 2015, Pearson, 2013, Prince et al., 2013).

PBS are self-control behaviours that can be spontaneously implemented by drinkers. Relying on individuals’ resources, PBS aim to reduces risky consumption and its negative consequences. Indeed, various studies have shown negative links between PBS use and consumption and consequences (Grazioli et al., 2015, Pearson, 2013, Prince et al., 2013). Furthermore, recent studies showed that psychosocial determinants of alcohol consumption (such as such as depression symptoms, drinking motives, perceived drinking norms, and drinking-related social identity) might be important psychological barriers to their use (Bravo et al., 2016, Bravo et al., 2019, Fleury et al., 2025, Linden et al., 2014, Patrick et al., 2011, Pearson, 2013, Scaglione et al., 2015). Among these determinants, drinking social norms have been demonstrated having a central role, influencing drinking habits (Mange et al., 2021). Norms are the rules that guide behaviours by encouraging conformity (Chung & Rimal, 2016). They can be injunctive, i.e., the perceived approval of the behaviour by peers; or descriptive, i.e., the perceived frequency of the behaviour among peers (Borsari & Carey, 2001). In complement to this norm compliance issue trough conformity, motivations to drink are also some of the main predictors of alcohol consumption (Bakkali et al., 2023, Lannoy et al., 2020) and barriers to PBS use. Enhancement motives capture drinking for fun and pleasure, social motives pertain to drinking to facilitate social interaction, and coping motives involve drinking to reduce negative affect (Cooper, 1994).

Conclusions from the current PBS literature - both on their effectiveness in reducing alcohol consumption and on psychological barriers to their use - remain limited in two key respects. First, although studies have consistently shown associations with reduced alcohol use and related harms (Prince et al., 2013), these findings are mostly correlational, as prior research on PBS determinants and barriers. Moreover, interventions designed to increase PBS use have shown mixed effectiveness on actual drinking behaviors (LaBrie et al., 2015, Peterson et al., 2021, Sugarman and Carey, 2009). Although many studies have relied on cross sectional designs, several longitudinal and cross-lagged investigations have examined reciprocal associations between PBS use and alcohol outcomes (e.g., Martens et al., 2011, Napper et al., 2014, Pearson et al., 2013, Treloar et al., 2015). However, findings remain heterogeneous across domains and modelling approaches. Across these studies, strategies aimed at modifying the manner of drinking (MOD) often emerge as protective, yet several longitudinal analyses report non-significant or mixed effects depending on the outcome and modelling approach. Recent systematic evidence likewise indicates variability rather than full convergence across PBS subscales (Cox et al., 2024). A structured overview of longitudinal PBS studies illustrating this heterogeneity is provided in Supplementary Material B.1.

Second, if traditionally PBS are categorised in three types - Serious Harm Reduction (SHR), Stopping/Limiting Drinking (SLD) and modifying the Manner Of Drinking (MOD) (Treloar et al., 2015) - recent studies have proposed alternative structural refinements. In this literature, Manner Of Drinking is variably abbreviated as MOD, MoD, or MD depending on the source; however, these labels all refer to the same subdimension of the PBSS/PBSS-20 framework (e.g., De Leon et al., 2022, Grazioli et al., 2019, Martens et al., 2007, Peterson et al., 2021, Treloar et al., 2015). Beyond these terminological variations, recent work has also questioned the underlying structure of PBS, with some studies suggesting a two-factor structure distinguishing direct/controlled consumption strategies from indirect/harm reduction strategies (Peterson et al., 2024). In parallel, factor-analytic work in European samples has questioned the internal coherence of the SLD dimension. Factor-analytic studies conducted in European samples suggest that SLD may encompass two conceptually distinct strategies: Mixing alcoholic with non-alcoholic drinks (MIX), and Planning Limits on Drinking (PLD) (e.g., Fleury et al., 2025, Grazioli et al., 2019). Distinguishing these strategies leads to a four-domain structure that may provide a more differentiated representation of protective behaviours. This refined four-factor structure may better capture variability in PBS use among European students. However, the psychosocial barriers that may hinder the adoption of these four strategies, as well as their specific protective dimension, have not been examined longitudinally within this refined framework. While prior longitudinal studies have investigated PBS using the traditional three-factor structure, these studies have primarily examined static associations, that is, whether individuals who report higher levels of certain psychosocial determinants also report higher levels of protective behavioral strategies or lower alcohol-related harm at later time points. Although informative, such approaches do not capture whether changes in psychosocial determinants are associated with corresponding changes in the use of protective strategies over time. To date, no longitudinal study to our knowledge has examined PBS effectiveness within this refined domain structure while explicitly modeling these dynamic change processes. In particular, no study has simultaneously examined dynamic associations between psychosocial determinants and changes in specific PBS domains - that is, whether changes in these determinants are associated with proportional changes in protective strategies over time - while also testing their prospective associations with alcohol outcomes within the same longitudinal framework. Dynamic longitudinal models such as Latent Change Score Models allow the examination of such change-to-change relationships between constructs (Kievit et al., 2018, McArdle, 2009). Addressing this gap is important because examining change-to-change relationships within a refined PBS domain structure may clarify which strategies are most dynamic and potentially most protective over time. If recent correlational research suggests that PLD and MOD strategies could be the most protective against risky drinking (Fleury et al., 2025), longitudinal findings remain variable across domains and contexts. Thus, longitudinal designs are needed to address these two concerns by examining how psychological barriers and alcohol consumption relate to PBS use over time.

### 1.1 Research overview

**Fig. 1:**
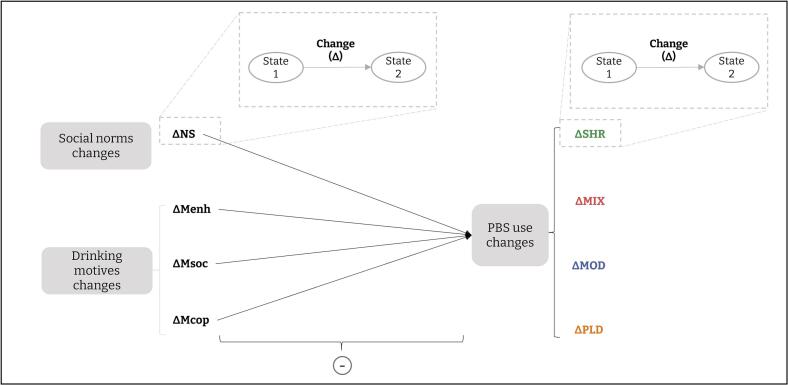
Latent Change Score Model hypotheses with PBS as outcomes and drinking social norms and motivations to drink as parameters. *Note.* Deltas (Δ) represent a change in each variable between T1 and T2. Each latent variable (each variable at a given time) implies a latent change model with auto-correlated errors.

Therefore, the first aim of this research was to examine the role of key psychosocial determinants of alcohol use (namely drinking motives, and perceived drinking social norms that favour) as potential barriers to the use of the four types of PBS. Study 1 addressed this first objective by testing in a two-wave Latent Change Score Model (LCSM, McArdle, 2009) if one-year changes in social norms and motivations to drink induce changes in each PBS use (see Fig. 1).

**Fig. 2:**
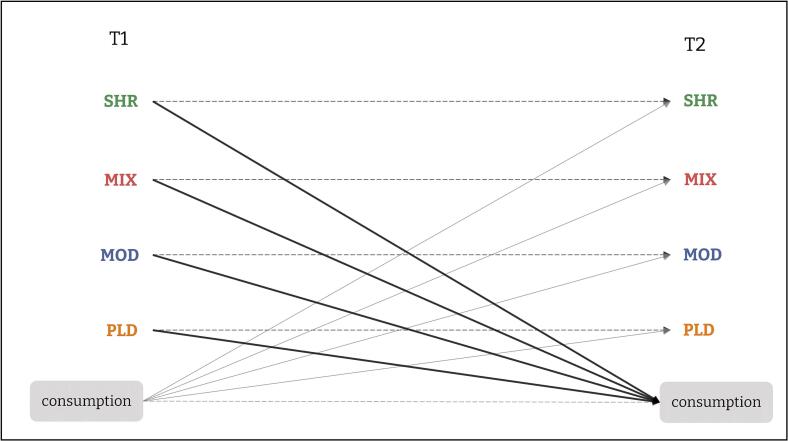
Cross-Lagged Panel Model hypotheses with alcohol consumption per typical occasion as outcome and PBS as predictors.*Note.* Covariances at T1 and residuals covariance at T2 will be calculated between all predictors. Solid arrows indicate hypothesized negative cross-lagged paths; dashed arrows indicate hypothesized positive autoregressive paths.

The second aim was to examine the prospective associations of the refined four‑PBS typology with risky drinking behaviors and alcohol‑related consequences. To address this objective, Study 2 employed a two-wave Cross-Lagged Panel Model (CLPM, Selig & Little, 2012), which tests whether PBS use at baseline is prospectively associated with drinking outcomes one year later, over and above their prior levels. The CPLM also enables an examination of temporal precedence between PBS use and consumption indicators (see Fig. 2).

## 2 Study 1

### 2.1 Method and participants

#### 2.1.1 Ethics

This study was authorized by the Research Ethics Committee of Caen University (France) with the registration n°2025051520474500000310000390 and preregistered at https://osf.io/xqtpc/overview before any access to, consultation of, or analysis of the database. Ethical approval covered the full study protocol, recruitment procedures, informed consent, and data management safeguards. All procedures and analytical plans were defined prior to examining the data.

#### 2.1.2 Participants and procedure

The participants were 164 volunteer university students who were contacted by email between September and November 2021, 2022 and/or 2023 through the university’s mailing list and invited to complete a computerized questionnaire using LimeSurvey (see Supplementary Material A.1. for all descriptive statistics, including measure of risky drinking, i.e., Alcohol Use Disorders Identification Test; Gache et al., 2005). Participants (*M*=19.80, *SD*=1.97) included 93 women and 68 men. All had consumed alcohol at least once in the past year of completion and completed entirely the PBS, social norms and drinking motivations scales over two consecutive years (80 participants answered in 2021-2022 and 84 in 2022-2023). Overall, participants showed low-to-moderate alcohol-related risk (*M_T1_*= 8.02, *SD_T1_*=6.06; *M_T2_*=8.34, *SD_T2_*=5.81).

### 2.2 Materials and measures

All items are available online at https://osf.io/g3y85/overview?view_only=.

#### 2.2.1 Protective Behavioral Strategies

*PBS use* in the past year were assessed using the PBSS-20 (Protective Behavioral Strategies Scale, Grazioli et al., 2019), a 20-item scale derived from the original scale by Martens et al. (2005, 2007). and its subsequent refinement by Treloar et al. (2015). Its psychometric properties have been examined in French-speaking samples using both confirmatory and exploratory approaches (Fleury et al., 2025, Grazioli et al., 2019). Using a 6-point Likert scale (from 1 = “Never” to 6 = “Always”), participants indicated how often they typically used each strategy when drinking alcohol.

After confirmatory factorial analyses (CFI_T1_=.928; TLI_T1_=.914; RMSEA_T1_=.052, 90%CI [.034; 0.69]; CFI_T2_=.916; TLI_T2_=.899; RMSEA_T2_=.058, 90%CI [.040; 0.74]), mean scores have been computed for each type (i.e., SHR, MIX, MOD and PLD strategies).

#### 2.2.2 Psychosocial determinants of alcohol consumption

Drinking Motives Questionnaire-Revised (DMQ-R; Cooper, 1994; 12 items) assessed *drinking motivations*, identifying social (α_T1_=.858 & α_T2_=.821), coping (α_T1_=.854 & α_T2_=.899) and enhancement (α_T1_=.802 & α_T2_=.732) motives.

Six items assessed *drinking social norms* (α_T1_=.687 & α_T2_=.917), three for injunctive norms (perceived approval of excessive drinking by peers), and others for descriptive norms (perceived frequency of excessive drinking among peers) (Borsari & Carey, 2001).

### 2.3 Statistical analyses

#### 2.3.1 Time invariance for internal PBS, norms and motives measures

Multigroup confirmatory factorial analyses (using the lavaan package in R Studio 2023.06.0 ; , 2020, Rosseel, 2012) tested time measurement invariance on the used measures through three nested models: configural, metric, and scalar invariance, compared sequentially (Steenkamp & Baumgartner, 1998). The aim was to determine if the constructs were measured equivalently across time and that observed differences reflected true change rather than changes in measurement properties, prior running longitudinal analyses. Invariance was assessed using CFI and RMSEA thresholds (Boateng et al., 2018, Cheung and Rensvold, 2002), with CFI differences ≤ 0.010 and RMSEA differences ≤ 0.015 between models (Mauduy et al., 2025). The Satorra-Bentler χ^2^ difference test was also applied, requiring non-significance (Satorra & Bentler, 2001). Invariance is supported if at least one criterion is met: non-significant χ^2^ difference, CFI_diff_≤0.010, or RMSEA_diff_≤0.015 (Mauduy et al., 2023).

#### 2.3.2 Longitudinal analysis of PBS use predictors

LCSM (Ghisletta & Mcardle, 2012) offer a longitudinal framework for assessing how within-person changes (symbolised with Δ) in one set of variables are coupled with changes in another set of variables over time. We estimated a single multivariate LCSM for the four PBS types (SHR, MIX, MOD, PLD). For each type, we specified the proportional-change path, which models change as a percentage of the previous score (i.e., the score at T2 is defined as a fixed proportion of the score at T1) and residual covariances among Δ factors were freely estimated. Changes in perceived norms and drinking motives (Δsocial, Δcoping, Δenhancement), plus age (centred at T1) and sex (coded -0.5 for men and +0.5 for women) as covariables, were entered as exogenous predictors of each Δ factor (ΔSHR, ΔMIX, ΔMOD, ΔPLD). Because PBS indicators and determinants were assessed on Likert-type scales and showed slight non-normality, we estimated the model using Robust Maximum Likelihood (MLR).

#### 2.3.3 Sensitivity analysis

Because no a priori power analysis was conducted prior to data collection, we conducted a Monte Carlo–based sensitivity analysis to evaluate statistical sensitivity of the Latent Change Score Model under conventional error rates (α=.05, two-tailed; target power=.80) for a 164 participants sample size. Following recommendations for structural equation modeling (MacCallum et al., 1996, Muthén and Muthén, 2002, Wolf et al., 2013), simulations were based on the final estimated model structure and parameter precision. For each structural regression, we estimated power at the observed standardized effect and calculated the minimum detectable standardized effect (β) required to reach the target power.

## 3 Results and discussion

### 3.1 Time invariance for internal PBS, norms and motives measures

Configurational, metric and scalar invariance all showed acceptable fit (see Table 1). No significant difference appeared between configurational and metric models (χ^2^_diff_=36.083; CFI_diff_=.003; RMSEA_diff_=0). Although metric and scalar models showed a significant difference, suggesting lack of partial measurement invariance, RMSEA difference remained acceptable (χ^2^_diff_=44.451, *p*=.007; CFI_diff_=.004; RMSEA_diff_=0), supporting time invariance for loadings and intercepts. Participants therefore have a similar understanding of PBS, motivations and norms at T1 and at T2.

**Table 1:** Model fit parameters for the three invariance models.

	Configural invariance	Metric invariance	Scalar invariance
Satorra-Bentler χ^2^ (df)	2759.126 (1800)	2795.112 (1824)	2839.664 (1848)
CFI	0.828	0.825	0.821
RMSEA [90%CI]	0.056 [.051; 0.060]	0.056 [.051; 0.060]	0.056 [.052; 0.060]

*Note. n* = 164; CFI = Comparative Fit Index; RMSEA = Root Mean Square Error of Approximation with 90% confidence interval.

### 3.2 Latent change score in norms and motivations on PBS use

Details of statistical results are displayed in Table 2.

**Table 2:** Multivariate Latent Change Score Model for PBS dimensions: proportional change and predictors of intra-individual change.

**Outcome**	Parameter	Estimate	SE	β	
**ΔSHR**	Proportional change	−0.498	0.051	−0.611	***
	ΔNorms	0.010	0.033	0.020	
	ΔSocial motives	0.111	0.059	0.118	†
	ΔCoping motives	−0.072	0.063	−0.077	
	ΔEnhancement motives	0.011	0.075	0.011	
	Age	−0.054	0.028	−0.124	*
	Sex	0.121	0.121	0.070	
**ΔMIX**	Proportional change	−0.379	0.067	−0.442	***
	ΔNorms	0.012	0.051	0.016	
	ΔSocial motives	−0.174	0.107	−0.121	
	ΔCoping motives	0.004	0.110	0.003	
	ΔEnhancement motives	−0.151	0.099	−0.099	
	Age	0.005	0.045	0.008	
	Sex	0.016	0.192	0.006	
**ΔMOD**	Proportional change	−0.292	0.060	−0.363	***
	ΔNorms	0.047	0.033	0.085	
	ΔSocial motives	−0.174	0.084	−0.169	*
	ΔCoping motives	−0.093	0.059	−0.090	
	ΔEnhancement motives	0.005	0.089	0.004	
	Age	0.084	0.034	0.174	*
	Sex	−0.287	0.142	−0.150	*
**ΔPLD**	Proportional change	−0.667	0.092	−0.580	***
	ΔNorms	0.042	0.053	0.053	
	ΔSocial motives	−0.109	0.103	−0.074	
	ΔCoping motives	0.065	0.086	0.045	
	ΔEnhancement motives	0.140	0.141	0.090	
	Age	−0.002	0.040	−0.003	
	Sex	0.023	0.175	0.008	

*Note.* β is the standardized estimate. Proportional change parameters correspond to autoregressive effects of PBS_T1 on ΔPBS (more negative values indicate stronger pull toward the mean). Sex is coded −0.5 for men and + 0.5 for women. Overall model fit: χ^2^(28) = 33.623, *p* = 0.214, CFI_robust_ = 0.990, TLI_robust_ = 0.972, RMSEA_robust_ = 0.034, 90%CI [.000; 0.076], SRMR = 0.056, AIC = 6602.840, BIC = 6884.927.

*** *p* < 0.001; ** *p* < 0.01; * *p* < 0.05; † *p* < 0.65.

The LCSM converged normally, showed acceptable overall fit, and explained a meaningful share of variance in the latent change factors: *R*^2^=.360 for ΔSHR, .231 for ΔMIX, .273 for ΔMOD, and .348 for ΔPLD. Residual covariances among the latent change factors were positive and small-to-moderate in magnitude, suggesting partially shared change processes across PBS domains.

At baseline, women scored higher on SHR and MOD (β=.314 and .292, both *p*<.001) and slightly higher on PLD (β=.165, *p*=.033) than men, whereas age was negatively related to SHR and PLD (β=−.193 and −.186; *p*=.009 and .004, respectively).

All proportional-change paths were negative and statistically significant, indicating a regression toward the mean of PBS use. This reveals convergence over time: individuals using PBS most of the time showed smaller subsequent gains (or slight declines), whereas those using less frequently PBS showed larger gains in their use. This pattern is also consistent with the age-related shifts we observed (MOD, rarely used, tended to increase with age while SHR, frequently used, tended to decrease).

**Fig. 3:**
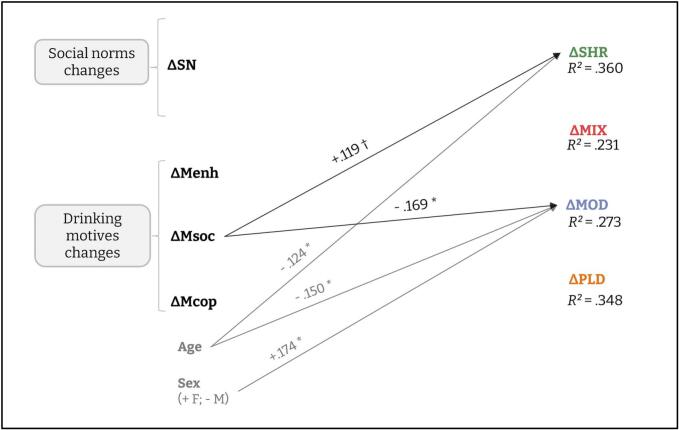
Latent Change Score Model results with PBS as outcomes and drinking social norms and motivations to drink as parameters. *Note.* Only significant links are represented, but the model tested links between all predictors and PBS. *** *p* < 0.001; ** *p* < 0.01; * *p* < 0.05; † *p* < 0.65.

Focusing on predictors of ΔPBS (change in PBS use), results showed that MOD is the most dynamic PBS. Higher age predicted positive changes in MOD strategies, men showed larger increased over time than women, and changes in social motives negatively predicted changes in MOD strategies: as social motives increased, the use of MOD decreased. For the other PBS, only age was a small negative predictor of changes in SHR strategies (ΔSHR) (see Fig. 3).

### 3.3 Sensitivity and interpretability

Monte Carlo sensitivity analyses were conducted under α=.05 (two-tailed) and target power=.80 to determine the range of effect sizes reliably detectable in the present design. Large proportional-change parameters (e.g., β_SHR_=−.611; β_PLD_=−.580) were well above the minimum detectable thresholds and can therefore be interpreted as robust convergence effects. For Δdeterminant → ΔPBS paths, sensitivity was lower. Notably, the significant association between changes in social motives and changes in MOD (β=−.169, *p*=.038) was detectable in the present sample, but falls below the standardized effect size corresponding to 80% power for some structural paths (all minimum detectable standardized effects and observed latent-changes estimates are reported in Supplementary Material A.2.). This indicates that although the effect reached statistical significance, its estimation is comparatively less precise and should be interpreted as a small-to-moderate change association rather than as a strong longitudinal determinant. Non-significant Δdeterminant → ΔPBS paths should be interpreted cautiously, as small change effects may have remained undetected.

Thus, although prior correlational work has identified both social norms and drinking motives as potential barriers to PBS use, these longitudinal results suggest that social drinking motives may constitute a psychological barrier to MOD use, although sensitivity analyses indicate that smaller change effects cannot be ruled out. Promoting MOD strategies – which seem to be particularly effective in reducing risky alcohol consumption (Fleury et al., 2025) – may therefore require interventions that directly target and attenuate social motives for drinking, which appear to work in opposition to this protective strategy. Study 2 now investigates whether PBS use prospectively reduces alcohol consumption and related consequences, and whether certain PBS types are more effective than others in doing so.

## 4 Study 2

### 4.1 Method and participants

#### 4.1.1 Ethics

This study was authorized by the Research Ethics Committee of Caen University (France) with the registration n°2025051520474500000310000390 and preregistered at https://osf.io/hz9xg/overview before any access to, consultation of, or analysis of the database. Ethical approval covered the full study protocol, recruitment procedures, informed consent, and data management safeguards. All procedures and analytical plans were defined prior to examining the data.

#### 4.1.2 Participants and procedure

The participants were 188 volunteer university students who were contacted by email between September and November 2021, 2022 and/or 2023 through the university’s mailing list and invited to complete a computerized questionnaire using LimeSurvey (see Supplementary Material A.3. for all descriptive statistics). Participants (*M*=19.90, *SD*=2.01) included 111 women and 74 men. All had consumed alcohol at least once in the past year of completion and completed entirely the PBS and alcohol consumption scales over 2 consecutive years (93 participants answered in 2021-2022 and 95 in 2022-2023).

### 4.2 Materials and measures

#### 4.2.1 Protective Behavioral Strategies

Identical to Study 1.

#### 4.2.2 Alcohol consumption

*Risky alcohol consumption* was assessed using the French version of the Alcohol Use Disorders Identification Test (AUDIT; Gache et al., 2005; α_T1_=.825 & α_T2_=.827), a 10-item tool that identifies excessive alcohol consumption and the risk of developing an AUD (Babor et al., 2001). The 10 AUDIT items cover three domains: hazardous alcohol use (consumption; items 1–3, i.e., drinking frequency, typical quantity, and heavy episodic drinking), dependence symptoms (items 4–6), and alcohol-related problems (items 7–10).

### 4.3 Statistical analyses

As in Study 1, multigroup confirmatory factorial analyses tested time measurement invariance for PBS and AUDIT measures.

#### 4.3.1 Cross-Lagged Panel Model

We fit a CLPM (Selig & Little, 2012) in R Studio (RStudio Team, 2020) with a latent AUDIT factor and four latent PBS types for examining reciprocal associations between these variables over time, namely whether prior PBS use predicts later alcohol consumption and, conversely, whether alcohol consumption predicts subsequent PBS use, over and above temporal stability. The structural model included autoregressive paths for each construct and cross-lagged paths in both directions; T1 latent covariances and T2 residual covariances were freely estimated using MLR. Consistent with recent methodological discussions, cross-lagged paths are interpreted as conditional longitudinal associations rather than as strictly within-person or causal effects (Lucas, 2023).

Although the preregistered analyses used the total AUDIT score, this composite combines heterogeneous components (consumption, dependence, harms) that are poorly aligned with the specific effects of PBS, which target concrete drinking behaviours rather than distal clinical outcomes. From a psychometric and theoretical perspective, prediction is improved when constructs are matched in terms of their level of specificity (Ajzen, 2005, Ajzen and Fishbein, 1977), and echoed in health behaviour research showing that conceptually aligned variables yield more valid estimates of behavioural change processes (Hagger et al., 2020). The AUDIT item assessing the typical number of drinks per typical occasion offered a more proximal, behaviourally specific criterion that aligns closely with the behaviours PBS aim to regulate (e.g., limiting drinks, alternating beverages). To enhance construct validity and clarify the behavioural impact of PBS, we conducted and only reported here an exploratory CLPM using this item to examine whether PBS use predicted subsequent drinking quantity. Results of the preregistered CLPM on AUDIT total score, and results of an exploratory CPLM on AUDIT-consequences subscore are available on respectively Supplementary Materials A.4. and A.5. They did not reveal any significant cross-lagged relationship, thus have not been included in the main part of this study.

#### 4.3.2 Sensitivity analyses

As no a priori power analysis was conducted, we performed Monte Carlo–based sensitivity analyses to determine the minimum detectable standardized cross-lagged effects under α=.05 (two-tailed) and target power=.80 for a 188 participants sample size. Simulations were conducted based on the final CLPM structure estimated with MLR. For each cross-lagged path, we derived the minimum detectable standardized effect (MDES) corresponding to 80% power.

## 5 Results and discussion

### 5.1 Time invariance for internal PBS and AUDIT measures

Configural, metric, and scalar invariance all showed acceptable fit (see Table 3). No significant difference appeared between configurational and metric models (χ^2^_diff_=30.485; CFI_diff_=0; RMSEA_diff_=.002), nor between metric and scalar models (χ^2^_diff_=17.760; CFI_diff_=.003; RMSEA_diff_=0), supporting time invariance. Participants therefore have a similar understanding of PBS and AUDIT at T1 and at T2.

**Table 3:** Model fit parameters for the three invariance models.

	Configural invariance	Metric invariance	Scalar invariance
Satorra-Bentler Χ^2^ (df)	1896.470 (1305)	1896.470 (1824)	1944.173 (1431)
CFI	0.869	0.869	0.866
RMSEA [90%CI]	0.049 [.044; 0.54	0.049 [.044; 0.054]	0.046 [.041; 0.051]

*Note. n* = 188; CFI = Comparative Fit Index; RMSEA = Root Mean Square Error of Approximation with 90% confidence interval.

### 5.2 Protective role of PBS against drinking

The CLPM showed acceptable fit (see Table 4), and explained a meaningful share of variance: corresponding latent *R*^2^ were .270 (consumption), .557 (SHR), .484 (MIX), .437 (MOD), and .252 (PLD).

**Table 4:** CPLM of bidirectional associations between alcohol use (item 2 of AUDIT, assessing the typical consumption in quantity of drinks) and PBS.

**Outcome (T2)**	Predictor (T1)	Path type	Estimate	SE	β		β 95%CI
**quantity**	SHR	PBS →Consumption	−0.022	0.137	−0.021		[-0.270; 0.229]
	MIX	PBS →Consumption	−0.047	0.086	−0.050		[-0.120; 0.227]
	MOD	PBS →Consumption	−0.315	0.127	−0.339	*	[-0.583; −0.096]
	PLD	PBS →Consumption	0.039	0.065	0.053		[-0.227; 0.128]
	quantity	Autoregressive	0.261	0.091	0.246	**	[0.077; 0.414]
**SHR**	SHR	Autoregressive	0.730	0.097	0.747	***	[0.572; 0.921]
	quantity	Consumption →PBS	0.001	0.081	0.001		[-0.164; 0.166]
**MIX**	MIX	Autoregressive	0.717	0.091	0.669	***	[0.527; 0.810]
	quantity	Consumption →PBS	−0.095	0.128	−0.061		[-0.221; 0.099]
**MOD**	MOD	Autoregressive	0.694	0.085	0.669	***	[0.509; 0.830]
	quantity	Consumption →PBS	0.021	0.080	0.018		[-0.114; 0.150]
**PLD**	PLD	Autoregressive	0.581	0.118	0.491	***	[0.304; 0.677]
	quantity	Consumption →PBS	−0.045	0.124	−0.034		[-0.217; 0.149]

*Note.* All variables shown in the table are latent variables. β is the standardized estimate. All paths are estimated while freely allowing covariances among latent variables at Time 1 and residual covariances among corresponding constructs at Time 2. Overall model fit: χ^2^(571) = 745.73, *p* < 0.001; CFI = 0.919; TLI = 0.910; RMSEA = 0.040, 90%CI[.032, 0.048]; SRMR = 0.068; AIC = 23061.14; BIC = 23485.12.

*** *p* < 0.001; ** *p* < 0.01; * *p* < 0.05.

**Fig. 4:**
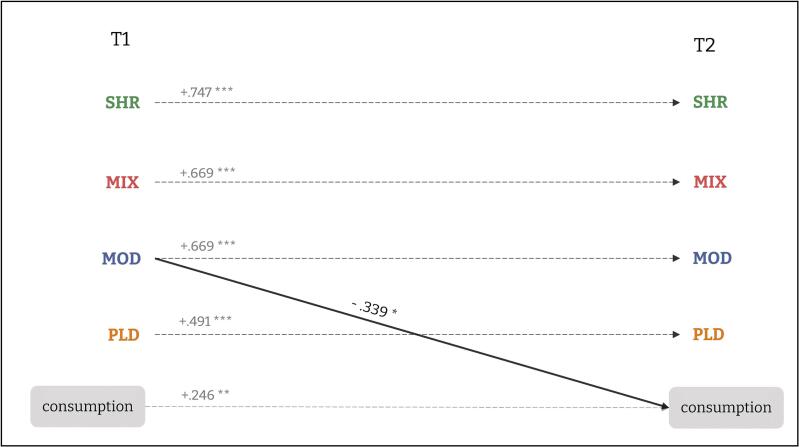
Cross-Lagged Panel Model results with alcohol consumption per typical occasion as outcome and PBS as predictors. *Note.* For reasons of readability, only significant links are represented, but the model tested links between all PBS and outcome, as well as covariances at T1 and residual covariances at T2. *** *p* < 0.001; ** *p* < 0.01; * *p* < 0.05.

Autoregressive stability varied, indicating strong carryover for PBS and a weaker carryover for the number of drinks per typical occasion (see Table 4). Globally, cross-lagged effects of PBS use (T1) on consumption (T2) are significant (global Wald χ^2^(4)=16.76, *p*=.002), whereas consumption (T1) did not predict later PBS (Wald χ^2^(4)=1.06, *p*=.900; see Table 4 for more details). This pattern suggests that certain PBS (particularly MOD strategies) may act as protective factors that are negatively associated with the number of drinks on a typical occasion, rather than being used more simply because individuals already drink less. More precisely, the only significant cross-lagged effect was MOD(T1) → consumption(T2), meaning that greater use of MOD strategies at baseline predicted lower subsequent quantity over and above the initial quantity of alcohol consumption (see Fig. 4). This result is consistent with a prospective protective association of MOD strategies: greater use at baseline predicted a lower number of drinks one year later, over and above initial drinking levels.

### 5.3 Sensitivity and interpretability

Monte Carlo analyses estimated the minimum detectable standardized cross-lagged effects (MDES) required to reach 80% power (α=.05). For PBS(T1) → consumption(T2) paths, MDES ranged between β=.35 and β=.65 depending on the structural path (all minimum detectable standardized effects and observed cross-lagged estimates are reported in Supplementary Material A.6.). The observed cross-lagged association MOD(T1) → consumption(T2) was β=−.339 (*p*=.013). This effect is statistically significant and therefore detectable in the present sample. However, it lies slightly below the MDES corresponding to 80% power for the MOD → consumption path (β=.650). Accordingly, this prospective association can be interpreted as a moderate effect detected with less-than-optimal power, meaning that suggesting a prospective association in this sample, its magnitude should be interpreted cautiously. For other PBS domains (SHR, MIX, PLD), cross-lagged estimates were substantially below their respective MDES values and non-significant; therefore, small prospective effects cannot be ruled out.

These findings suggest that while MOD strategies show a longitudinal association with subsequent drinking quantity, conclusions regarding other PBS domains remain tentative given the limited sensitivity of the design to detect small longitudinal effects. Same-wave associations between PBS domains and number of drinks were examined through covariances at T1 and residual covariances at T2. At T1, number of drinks correlated negatively with all PBS domains, consistent with concurrent links where higher PBS co-occurred with lower quantity of drinks; at T2, a residual negative covariance remained between number of drinks and MOD, indicating a same-wave association in the same direction (all T1 and T2 covariances are shown in Table 5). This pattern is consistent with PBS use characterising a lower-risk drinking style at a given wave, even after temporal stability and cross-lagged paths are taken into account.

**Table 5:** Latent covariances and residual covariances in the CPLM of typical consumption and PBS.

**Time**	Variable 1	Variable 2	Estimate	SE	β		β 95%CI
**T1**	Consumption	SHR	−0.257	0.121	−0.294	*	[-0.493; −0.020]

		MIX	−0.509	0.125	−0.389	***	[-0.754; −0.263]
		MOD	−0.505	0.108	−0.500	***	[-0.717; −0.294]
		PLD	−0.289	0.099	−0.291	**	[-0.484; −0.094]
	SHR	MIX	0.600	0.170	0.476	***	[0.267; 0.933]
		MOD	0.632	0.134	0.632	***	[0.369; 0.894]
		PLD	0.550	0.142	0.561	***	[0.272; 0.828]
	MIX	MOD	0.760	0.145	0.520	***	[0.475; 1.045]
		PLD	0.536	0.146	0.373	***	[0.250; 0.822]
MOD	PLD	0.587	0.121	0.516	***	[0.350; 0.823]
**T2**	Consumption	SHR	0.010	0.053	0.020		[-0.094; 0.115]
		MIX	−0.048	0.075	−0.054		[-0.196; 0.099]
		MOD	−0.133	0.062	−0.186	*	[-0.255; −0.011]
		PLD	−0.021	0.068	−0.023		[-0.155; 0.113]
	SHR	MIX	0.325	0.151	0.514	*	[0.029; 0.622]
		MOD	0.287	0.126	0.568	*	[0.040; 0.534]
		PLD	0.349	0.134	0.534	**	[0.086; 0.611]
	MIX	MOD	0.423	0.128	0.482	**	[0.173; 0.674]
		PLD	0.327	0.134	0.289	*	[].064; 0.589
	MOD	PLD	0.458	0.120	0.506	***	[0.223; 0.693]

*Note.* β is the standardized estimate. Data show covariances among latent AUDIT and PBS dimensions at Time 1 and residual covariances among the corresponding latent variables at Time 2. Covariances are freely estimated in the cross-lagged panel model including a latent factor (number of drinks) and four latent PBS dimensions (SHR, MIX, MOD, PLD).

*** *p* < 0.001; ** *p* < 0.01; * *p* < 0.05.

## 6 General discussion

This research aimed (1) to examine the role of drinking motives and perceived drinking social norms as barriers the four PBS use, through a Latent Change Score Model, and (2) to determine to what extent the use of each PBS reduces risky drinking behaviors among students, through a Cross-Lagged Panel Model. Study 1 showed that MOD emerged as the most dynamic Protective Behavioral Strategy: as age increases and social motives decrease, MOD use tends to increase over time. Study 2 showed that MOD in particular was longitudinally associated with a lower number of drinks one year later, although prior levels of consumption remained the strongest predictor of subsequent drinking. Importantly, sensitivity analyses indicate that this prospective association was detected in a design primarily sensitive to moderate longitudinal effects; its magnitude should therefore be interpreted cautiously and confirmed in larger longitudinal samples.

Modifying the manner of drinking (MOD) is characteristic of inhibiting risky consumption behaviours. Previously shown negatively linked to consumption indicators more than other PBS (e.g., Braitman et al., 2023, Martens et al., 2005) or characteristic to low drinkers (Fleury et al., 2025), this strategy is associated with fewer drinks consumed, and may therefore contribute to lower risk of binge drinking. Across all models, same-wave negative links between PBS and consumption were consistent with prior evidence that higher PBS co-occur with fewer consequences or lower consumption (Cox et al., 2024, Martens et al., 2004), but only MOD effects were strong enough to remain detectable one year later. Although the magnitude of this effect lies below the threshold corresponding to 80% power, its statistical significance indicates a prospective association that is unlikely to be null, while its size should be interpreted with caution. However, the absence of significant cross-lagged effects for other PBS domains should not be interpreted as definitive evidence of no longitudinal impact, as smaller prospective effects may not have been detectable within the present design.

One-year changes (an increase) in social drinking motives (drinking to facilitate social interactions and obtain social rewards) predicted changes (a decrease) in modifying the Manner Of Drinking. This is consistent with the idea that such motives foster participation in group-based, high-intensity drinking formats that MOD explicitly discourages (Freichel et al., 2023). Other determinants showed no unique longitudinal association with this strategy: perceived norms may contribute little once social motives are included, as both partly capture expectations of heavy social drinking, whereas coping and enhancement motives, which refer to regulating internal states and are relatively stable, may show limited within-person covariation with change in MOD over a one-year interval (Loose et al., 2018, Stevenson et al., 2019). More broadly, this research focused on drinking-related determinants as potential barriers to PBS use; future studies should extend these findings by identifying other protective factors and more direct predictors of PBS implementation, including students’ value-based appraisals of strategies (e.g., perceived costs and benefits and the extent to which these strategies fit their desired drinking identity and goals).

Furthermore, over one year, students who already used more strategies at baseline tended to change less, whereas those who used fewer PBS showed greater increases, indicating convergence in PBS use. This suggest that strategies can be adopted by initially low users rather than being only a stable drinking style. Age was negatively related to Serious Harm Reduction use at baseline, while higher age predicted greater increases in MOD over time. Taken together, these age effects suggest that as students grow older, they may less often expose to high-risk drinking situations requiring serious-harm reduction strategies and rely more on MOD strategies. Also, at baseline, women reported higher PBS than men, consistent with previous findings (e.g., Tabernero et al., 2019, Walters et al., 2007).

## 7 Limitations

This research has several limitations. First, both studies used a two-wave longitudinal design; as a result, causal interpretations and time-ordering should be viewed with caution. Although this design goes beyond cross-sectional work by allowing the examination of prospective associations, it does not provide causal evidence and does not allow a clear separation of within-person change from stable between-person differences. This limitation is particularly relevant for the Cross-Lagged Panel Model used in Study 2, as two-wave CLPM may reflect stable between-person differences rather than dynamic within-person processes. Nevertheless, the present design still allows the examination of prospective longitudinal associations between PBS use and drinking behaviours while accounting for prior levels of each construct, which represents an empirical step beyond the predominantly cross-sectional evidence currently available in this research area. In addition, potential reciprocal influences and unmeasured confounding cannot be fully ruled out. A third measurement wave and a larger sample would help to better disentangle trait-like stability from within-person change and to strengthen inferences about reciprocal effects and developmental trajectories. Nevertheless, examining these prospective associations still provides informative evidence regarding how distinct PBS domains relate to subsequent drinking behaviour. In particular, the present results add longitudinal support to prior findings highlighting the potential protective role of MOD, while also extending this literature by testing these associations within a refined four-domain framework and in a French student context. Second, the interval between waves was one year, which is long enough for changes in drinking habits and PBS use to consolidate. Designs with shorter lags (e.g., monthly) and multiple waves would likely capture additional dynamics and might reveal the role of other predictors or strategies types at a finer time scale. In addition, no a priori power analysis was conducted. Monte Carlo post-hoc sensitivity analyses indicate that the present longitudinal designs were primarily capable of detecting moderate effects. Consequently, small-to-moderate longitudinal associations may have been estimated with limited precision. Future studies with larger samples would allow more precise estimation of smaller dynamic effects and stronger conclusions regarding the comparative effectiveness of PBS domains. Third, all key constructs were assessed via self-report in French university students, which may limit the generalisability of our findings to other age groups, cultural contexts, and assessment methods. Drinking norms, prevention contexts, and PBS patterns may differ across countries; therefore, the present findings should be interpreted as context-specific and require replication in other cultural settings. At the same time, longitudinal evidence on PBS remains largely concentrated in North American samples; examining these associations in a French university context contributes to extending this literature across cultural settings.

## 8 Conclusion

From a preventive perspective, findings from these two longitudinal studies suggest that not all PBS are equivalent targets. Across both studies, strategies aimed at modifying the manner of drinking (i.e., inhibiting risky modes of consumption) consistently stood out: higher MOD use was associated with fewer drinks per occasion one year later. Social drinking motives emerged as a barrier to MOD, suggesting that interventions should also address the appeal of socially driven heavy drinking. In practical terms, student-focused programmes could prioritise messages that reframe MOD as compatible with enjoyment and social connection rather than as “spoiling the fun”. Before searching for highly specific, strategy-unique determinants, these results indicate that well-known levers such as motives and norms can already be mobilised to strengthen MOD use, which currently appears to be a particularly promising PBS target for reducing risky drinking among students.

## Declaration of generative AI and AI-assisted technologies in the writing process

During the preparation of this work, the authors used ChatGPT (GPT-5 version) in order to assist with the rephrasing of isolated sentences to improve their readability. After using this tool, the authors reviewed and edited the content as needed and take full responsibility for the content of the published article.

## CRediT authorship contribution statement

**Maëlle Fleury:** Writing – review & editing, Writing – original draft, Visualization, Validation, Project administration, Methodology, Formal analysis, Conceptualization. **Jessica Mange:** Writing – review & editing, Writing – original draft, Visualization, Validation, Project administration, Methodology, Funding acquisition, Conceptualization. **Maxime Mauduy:** Writing – review & editing, Writing – original draft, Visualization, Validation, Supervision, Project administration, Methodology, Formal analysis, Conceptualization.

## Declaration of competing interest

The authors declare that they have no known competing financial interests or personal relationships that could have appeared to influence the work reported in this paper.

## Data Availability

Databases have been shared with anonymised access links have been added in the manuscript for peer review. The non-anonymised preregistration is available at: https://osf.io/g3y85/registrations.
