# Supplementary material for: Longitudinal examination of a refined four-factor model of Protective Behavioural Strategies: Psychosocial barriers to their use and protective effects on students’ alcohol consumption
Source: Addict Behav Rep. 2026 Apr 3;23:100694. doi: 10.1016/j.abrep.2026.100694 (PMC13089060; doi:10.1016/j.abrep.2026.100694)
Supplement: Supplementary Data 1 [file mmc1.docx]

|  | **T1** | | **T2** | |
| --- | --- | --- | --- | --- |
|  | **Mean** | ***SD*** | **Mean** | ***SD*** |
| AUDIT total score | 8.02 | *6.057* | 8.34 | *5.809* |
| Social norms total score | 5.53 | *0.906* | 4.46 | *1.563* |
| Social motivations to drink | 8.91 | *3.325* | 9.11 | *3.207* |
| Coping motivations to drink | 5.82 | *3.095* | 5.74 | *3.111* |
| Enhancement motivations to drink | 9.20 | *3.327* | 9.13 | *3.098* |
| Use of SHR strategy | 4.80 | *1.055* | 4.83 | *0.912* |
| Use of MIX strategy | 4.24 | *1.545* | 4.15 | *1.536* |
| Use of MOD strategy | 3.56 | *1.178* | 3.53 | *1.205* |
| Use of PLD strategy | 2.78 | *1.175* | 2.63 | *1.202* |

**Supplementary Material A.1.** Descriptive statistics of used variables in Study 1

*Note.* 164 participants; SD = standard deviation; SHR = Serious Harm Reduction strategy; MIX = Mixing alcoholic with non-alcoholic drinks; MOD = modifying the Manner Of Drinking; PLD = Planning Limits on Drinking.

**Supplementary Material A.2.** Standardised effects and minimum detectable effects in Study 1

| **Outcome (T2)** | **Predictor**  **(T1)** | **Std. β** | ***SE*** | ***p-value*** | **95% CI** | **MDES (β)** |
| --- | --- | --- | --- | --- | --- | --- |
| **ΔSHR** | ΔNorms | 0.020 | 0.033 | 0.760 | [-0.054 ; 0.074] | 0.187 |
|  | ΔSocial motives | 0.118 | 0.059 | 0.062 | [-0.005 ; 0.227] | 0.200 |
|  | ΔCoping motives | -0.077 | 0.063 | 0.250 | [-0.195 ; 0.051] | 0.200 |
|  | ΔEnhancement motives | 0.011 | 0.075 | 0.887 | [-0.136 ; 0.157] | 0.200 |
|  | Age | -0.124 | 0.028 | 0.050 | [-0.109 ; 0.000] | 0.187 |
|  | Sex | 0.070 | 0.121 | 0.316 | [-0.116 ; 0.358] | 0.187 |
| **ΔMIX** | ΔNorms | 0.016 | 0.051 | 0.812 | [-0.087 ; 0.111] | 0.200 |
|  | ΔSocial motives | -0.121 | 0.107 | 0.102 | [-0.383 ; 0.035] | 0.237 |
|  | ΔCoping motives | 0.003 | 0.110 | 0.968 | [-0.211 ; 0.220] | 0.200 |
|  | ΔEnhancement motives | -0.099 | 0.099 | 0.127 | [-0.345 ; 0.043] | 0.200 |
|  | Age | 0.008 | 0.045 | 0.906 | [-0.082 ; 0.093] | 0.212 |
|  | Sex | 0.006 | 0.192 | 0.932 | [-0.359 ; 0.392] | 0.200 |
| **ΔMOD** | ΔNorms | 0.085 | 0.033 | 0.153 | [-0.017 ; 0.111] | 0.200 |
|  | ΔSocial motives | -0.169 | 0.084 | 0.038 | [-0.338 ; -0.010] | 0.225 |
|  | ΔCoping motives | -0.090 | 0.059 | 0.114 | [-0.209 ; 0.022] | 0.212 |
|  | ΔEnhancement motives | 0.004 | 0.089 | 0.956 | [-0.169 ; 0.179] | 0.225 |
|  | Age | 0.174 | 0.034 | 0.013 | [0.018 ; 0.150] | 0.185 |
|  | Sex | -0.150 | 0.142 | 0.043 | [-0.565 ; -0.008] | 0.200 |
| **ΔPLD** | ΔNorms | 0.053 | 0.053 | 0.430 | [-0.062 ; 0.146] | 0.187 |
|  | ΔSocial motives | -0.074 | 0.103 | 0.289 | [-0.311 ; 0.092] | 0.212 |
|  | ΔCoping motives | 0.045 | 0.086 | 0.448 | [-0.104 ; 0.234] | 0.200 |
|  | ΔEnhancement motives | 0.090 | 0.141 | 0.321 | [-0.137 ; 0.417] | 0.212 |
|  | Age | -0.003 | 0.040 | 0.962 | [-0.081 ; 0.077] | 0.200 |
|  | Sex | 0.008 | 0.175 | 0.897 | [-0.321 ; 0.366] | 0.200 |

Note. Δ denotes latent change scores estimated in the Latent Change Score Model(LCSM; i.e., intra-individual change from T1 to T2), 164 participants. Std. β corresponds to fully standardized estimates (std.all). 95% confidence intervals are reported for the unstandardized estimates. MDES = minimum detectable standardized effect required to achieve 80% power (α=.05), derived from Monte Carlo sensitivity analyses, simulations based on the final LCSM specification, estimator = MLR (FIML)

**Supplementary Material A.3.** Descriptive statistics of used variables in Study 2.

|  | **T1** | | **T2** | |
| --- | --- | --- | --- | --- |
|  | **Mean** | ***SD*** | **Mean** | ***SD*** |
| AUDIT total score | 8.04 | *6.085* | 8.24 | *6.042* |
| AUDIT consumption subscore | 4.42 | *2.179* | 4.47 | *2.087* |
| AUDIT dependency subscore | 1.14 | *1.728* | 1.19 | *1.775* |
| AUDIT consequences subscore | 2.48 | *3.084* | 2.58 | *3.043* |
| Use of SHR strategy | 4.80 | *1.012* | 4.81 | *0.964* |
| Use of MIX strategy | 4.28 | *1.475* | 4.11 | *1.556* |
| Use of MOD strategy | 3.67 | *1.181* | 3.58 | *1.215* |
| Use of PLD strategy | 2.82 | *1.139* | 2.66 | *1.229* |

*Note.* 188 participants; SD = standard deviation; SHR = Serious Harm Reduction strategy; MIX = Mixing alcoholic with non-alcoholic drinks; MOD = modifying the Manner Of Drinking; PLD = Planning Limits on Drinking.

**Supplementary Material A.4.** Longitudinal analysis of PBS use effect on risky consumption (AUDIT total score)

Despite overall acceptable fit for the CLPM (χ²(1361) = 1956.06, *p* < .001; CFI = .848; TLI = .840; RMSEA = .048, 90%CI[.043, .053]; SRMR = .076), cross-lagged paths between AUDIT and the four PBS domains were small and non-significant in both directions (Wald tests: PBS→AUDIT χ²(4) = 1.18, *p* = .882; AUDIT→PBS χ²(4) = 2.43, *p* = .657). Autoregressive stability was moderate to high (β: AUDIT = .845; SHR = .725; MOD = .666; MIX = .636; PLD = .480; all *p* < .001), suggesting that both AUDIT scores and PBS usage remained stable from a year to another (each construct was largely predicted by its own prior level). Latent *R*² were .745 (AUDIT), .559 (SHR), .499 (MIX), .446 (MOD), and .253 (PLD), highlighting a strong intra-individual continuity. Residual covariances at T2 showed several positive PBS interrelations and a small negative AUDIT–MOD association (*p* = .025).

**Supplementary Material A.5.** Exploratory analyses: protective role of PBS against consequences (AUDIT subscore)

Because the global AUDIT score relies on 3 aspects of drinking (i.e., consumption, dependency and consequences; Babor et al., 2001), and some strategies are supposed to reduce specifically one of these aspects (i.e., SHR), it could explain the lack of results. Thus, we ran again the CLPM with the 4 PBS and the subscore of AUDIT that accounts for consequences (regrets, blackouts, injuries and peers worrying).

Despite acceptable fit for the CLPM (χ²(797) = 1053.39, *p* < .001; CFI = .897; TLI = .889; RMSEA = .041; SRMR = .072; AIC = 26241.34; BIC = 26720.33), cross-lagged paths were small and non-significant in both directions (Wald: PBS(T1)→CONSEQ(T2) χ²(4) = 1.67, *p* = .796; CONSEQ(T1)→PBS(T2) χ²(4) = 1.15, *p* = .886). Autoregressive paths were moderate–high (β: CONSEQ = .920; SHR = .728; MOD = .643; MIX = .681; PLD = .476; all *p* < .001), indicating strong temporal stability. Latent R² were .783 (CONSEQ), .560 (SHR), .445 (MOD), .489 (MIX), and .247 (PLD), quantifying the variance explained at T2 by prior levels and covariates. At T1, AUDIT-consequences subscore correlated negatively with SHR, MIX, and MOD (all *p* ≤ .002; PLD ns). At T2, the residual covariance between consequences and MIX was small and negative (*p* = .034), a weak same-wave link in the same direction.

**Supplementary Material A.6.** Cross-Lagged standardised effects and minimum detectable effects in Study 2

| **Outcome**  **(T2)** | Predictor  (T1) | Std. β | SE | p-value | 95% CI | MDES (β) |
| --- | --- | --- | --- | --- | --- | --- |
| **Quantity** | SHR | −.021 | .137 | .872 | [−.270 ; .229] | .400 |
| **Quantity** | MIX | .053 | .065 | .547 | [−.120 ; .227] | .350 |
| **Quantity** | MOD | −.339 | .127 | .013 | [−.583 ; −.096] | .650 |
| **Quantity** | PLD | −.050 | .086 | .582 | [−.227 ; .128] | .388 |
| **SHR** | Quantity | .049 | .090 | .588 | [−.128 ; .226] | .213 |
| **MIX** | Quantity | .068 | .083 | .413 | [−.095 ; .231] | .200 |
| **MOD** | Quantity | −.017 | .086 | .843 | [−.185 ; .151] | .225 |
| **PLD** | Quantity | .063 | .081 | .440 | [−.095 ; .222] | .213 |

*Note.* CLPM = Cross-Lagged Panel Model, 188 participants; Std. β corresponds to fully standardized estimates (std.all). 95% confidence intervals are reported for the unstandardized estimates. MDES = minimum detectable standardized effect required to achieve 80% power (α=.05), derived from Monte Carlo sensitivity analyses, simulations based on the final CLPM specification, estimator = MLR (FIML).
