# Supplementary material for: Longitudinal examination of a refined four-factor model of Protective Behavioural Strategies: Psychosocial barriers to their use and protective effects on students’ alcohol consumption
Source: Addict Behav Rep. 2026 Apr 3;23:100694. doi: 10.1016/j.abrep.2026.100694 (PMC13089060; doi:10.1016/j.abrep.2026.100694)
Supplement: Supplementary Data 2 [file mmc2.docx]

**Supplementary Material B.1.** Summarize of main results of 16 longitudinal studies on PBS effects.

| **Reference** | **PBS measure** | **Results on MOD** | | ***Additional results*** |
| --- | --- | --- | --- | --- |
|  |  | **Consumption** | **Consequences** |  |
| **Dekker et al. (2018, 2020)** | Items mostly derived from Australian Institute of Health and Welfare (AIHW, 2021) | Non applicable | Non applicable | *Only "count your drinks" item was associated with a decrease in consumption* |
| **Dvorak et al. (2015)** | PBSS - 3 dimensions | Negative associations (only when the intervention shows a congruent framing with norms, as low norms for PBS use must be associated with positive framing and inversely for high norms) | | *No effect found for SLD and SHR* |
| **Fernández-Calderón et al. (2021)** |  | Negative associations (strongest predictor) on amount of alcohol, excessive consumption | Negative associations (strongest predictor) | *SHR was longitudinally related to lower frequency of alcohol consumption, SLD was not associated with any outcomes, and total PBS score was associated with all study outcomes.* |
| **Grazioli et al. (2015)** | PBSS - 3 dimensions | Negative association | Negative association | *SHR shows mixed effects on consequences (depends if positive and negative alcohol expectancies are low or high)* |
| **Grazioli et al. (2015b)** | PBSS - 3 dimensions | No significant association | No significant association | *No PBS were associated with consumption, SLD was associated with consequences => "mixed and unstable results"* |
| **Howard et al. (2024)** | PBSS - 3 dimensions | PBS do not clearly reduce consumption or consequences. | | |
| **Lewis et al. (2012)** | PBSS - 3 dimensions | Negative associations (within- and between-person) | Negative associations (within- and between-person) | *SHR show positive associations with blood alcohol concentration, number of drinks* |
| **Linden-Carmichel et al. (2015)** | Protective Behavioural Strategies Measure (PBSM) – 22 items (Novik & Boekeloo, 2011). Dichotomous daily version for follow-up, average score per day of consumption. | Non applicable | Non applicable | *PBS use is negatively linked to consumption (quantity, frequency and peak)* |
| **Martens et al. (2011)** | PBSS - 3 dimensions | No significant association | No significant association | *SLD decreases consumption but increases consequences in long term perspective, SHR decreases consequences* |
| **Napper et al. (2014)** | PBSS - 3 dimensions | Negative association | Negative association | *SHR reduces consequences, SLD shows now significant* association |
| **Pearson et al. (2013)** | PBSS - 3 dimensions | No significant association | Negative association | *SLD shows no association, SHR shows positive associations with both consumption and consequences* |
| **Peterson et al. (2021)** | Literature review listing 15 instruments, including: PBSS / PBSS-R / PBSS-20, PSQ, PBSM, SQ, PBSP, GPBSS, etc. | Globally negative association | Non applicable | *SLD globally decreases consumption whereas SHR globally decreses consequences* |
| **Richards et al. (2025)** | PBSS-20 but only SHR items were used | Non applicable | Non applicable | *SHR reduces damage even in cases of extreme consumption* |
| **Schultz et al. (2024)** | PBSS - 3 dimensions | No significant association | Some MOD item predict fewer consequences: avoiding drinking games, drinking slowly. Some MOD items (avoiding following others, avoiding mixing alcohols) predict more consequences | *Total PBS score → fewer consequences at 3 months. The majority of PBS have no significant* association*.* |
| **Treloar et al. (2015)** | PBSS-20 - 3 dimensions | No significant association | No significant association | *In the multivariate prospective model (controlling for baseline level and other subscales), SHR is the only subscale negatively related to consequences.* |

**References:**

Dekker, M. R., Jongenelis, M. I., Hasking, P., Kypri, K., Chikritzhs, T., & Pettigrew, S. (2020). Factors Associated with Engagement in Protective Behavioral Strategies among Adult Drinkers. Substance Use & Misuse, 55(6), 878–885. https://doi.org/10.1080/10826084.2019.1708944

Dekker, M. R., Jongenelis, M. I., Wakefield, M., Kypri, K., Hasking, P., & Pettigrew, S. (2018). A longitudinal examination of protective behavioral strategies and alcohol consumption among adult drinkers. Addictive Behaviors, 87, 1–7. https://doi.org/10.1016/j.addbeh.2018.06.017

Dvorak, R. D., Pearson, M. R., Neighbors, C., & Martens, M. P. (2015). Fitting In and Standing Out: Increasing the Use of Alcohol Protective Behavioral Strategies with a Deviance Regulation Intervention. Journal of Consulting and Clinical Psychology, 83(3), 482–493. https://doi.org/10.1037/a0038902

Fernández-Calderón, F., González-Ponce, B. M., Díaz-Batanero, C., & Lozano-Rojas, Ó. M. (2021). Predictive Utility of Protective Behavioral Strategies for Alcohol-Related Outcomes in a Community Sample of Young Adults. Journal of Studies on Alcohol and Drugs, 82(4), 476–485. https://doi.org/10.15288/jsad.2021.82.476

Grazioli, V. S., Dillworth, T., Witkiewitz, K., Andersson, C., Kilmer, J. R., Pace, T., Fossos-Wong, N., Carroll, H., Berglund, M., Daeppen, J.-B., & Larimer, M. E. (2015b). Protective behavioral strategies and future drinking behaviors: Effect of drinking intentions. Psychology of Addictive Behaviors, 29(2), 355–364. https://doi.org/10.1037/adb0000041

Grazioli, V. S., Lewis, M. A., Garberson, L. A., Fossos-Wong, N., Lee, C. M., & Larimer, M. E. (2015). Alcohol Expectancies and Alcohol Outcomes: Effects of the Use of Protective Behavioral Strategies. Journal of Studies on Alcohol and Drugs, 76(3), 452–458. https://doi.org/10.15288/jsad.2015.76.452

Howard, A. L., Lamb, M., Alexander, S. M., Bradley, A. H. M., Carnrite, K. D., Milyavskaya, M., Barker, E. T., & Patrick, M. E. (2024). Planned and unplanned drinking to get drunk: A registered report examining willingness, drinking motives, and protective behavioral strategies using ecological momentary assessment. Psychology of Addictive Behaviors, 38(5), 519–539. https://doi.org/10.1037/adb0000909

Lewis, M. A., Patrick, M. E., Lee, C. M., Kaysen, D. L., Mittman, A., & Neighbors, C. (2012). Use of Protective Behavioral Strategies and their Association to 21st Birthday Alcohol Consumption and Related Negative Consequences: A Between- and Within-person Evaluation. Psychology of Addictive Behaviors, 26(2), 179–186. https://doi.org/10.1037/a0023797

Linden-Carmichael, A. N., Braitman, A. L., & Henson, J. M. (2015). Protective Behavioral Strategies as a Mediator Between Depressive Symptom Fluctuations and Alcohol Consumption: A Longitudinal Examination Among College Students. Journal of Studies on Alcohol and Drugs, 76(1), 80–88. https://doi.org/10.15288/jsad.2015.76.80

Martens, M. P., Martin, J. L., Littlefield, A. K., Murphy, J. G., & Cimini, M. D. (2011). Changes in protective behavioral strategies and alcohol use among college students. Drug and Alcohol Dependence, 118(2), 504–507. https://doi.org/10.1016/j.drugalcdep.2011.04.020

Napper, L. E., Kenney, S. R., Lac, A., Lewis, L. J., & LaBrie, J. W. (2014). A cross-lagged panel model examining protective behavioral strategies: Are types of strategies differentially related to alcohol use and consequences? Addictive Behaviors, 39(2), 480–486.

Pearson, M. R., D’Lima, G. M., & Kelley, M. L. (2013). Daily Use of Protective Behavioral Strategies and Alcohol-Related Outcomes Among College Students. Psychology of Addictive Behaviors : Journal of the Society of Psychologists in Addictive Behaviors, 27(3), 10.1037/a0032516. https://doi.org/10.1037/a0032516

Peterson, R., Kramer, M. P., Pinto, D., De Leon, A. N., Leary, A. V., Marin, A. A., Cora, J. L., & Dvorak, R. D. (2021). A comprehensive review of measures of protective behavioral strategies across various risk factors and associated PBS-related interventions. Experimental and Clinical Psychopharmacology, 29(3), 236–250. https://doi.org/10.1037/pha0000498

Richards, V. L., Turrisi, R. J., Glenn, S. D., Mallett, K. A., Altstaedter, A., Ackerman, S., & Russell, M. A. (2025). Serious harm reduction protective behavioral strategies reduce consequences associated with alcohol-induced blackouts in college students. Addictive Behaviors, 162, 108234. https://doi.org/10.1016/j.addbeh.2024.108234

Schultz, N. R., Smith-LeCavalier, K. N., Walukevich-Dienst, K., Prince, M. A., & Larimer, M. E. (2024). Longitudinal examination of alcohol use motives, item-level protective behavioral strategies, and alcohol-related consequences. Alcohol, Clinical and Experimental Research, 48(4), 715–728. https://doi.org/10.1111/acer.15282

Treloar, H., Martens, M. P., & McCarthy, D. M. (2015). The Protective Behavioral Strategies Scale-20: Improved content validity of the Serious Harm Reduction subscale. Psychological Assessment, 27(1), 340–346. https://doi.org/10.1037/pas0000071
